# Complete Genome Sequence of *Methylosinus* sp. Strain C49, a Methane-Oxidizing Bacterium Harboring *phaABC* Genes for Polyhydroxyalkanoate Synthesis

**DOI:** 10.1128/MRA.00113-20

**Published:** 2020-07-02

**Authors:** Shohei Yasuda, Toshikazu Suenaga, Akihiko Terada

**Affiliations:** a Department of Chemical Engineering, Tokyo University of Agriculture and Technology, Koganei, Tokyo, Japan; b Global Innovation Research Institute, Tokyo University of Agriculture and Technology, Fuchu, Tokyo, Japan; University of Southern California

## Abstract

We report a complete genome sequence of *Methylosinus* sp. strain C49, a methane-oxidizing bacterium (MOB) in the class *Alphaproteobacteria*, isolated from MOB-enriched biomass. The genome encodes the functional genes for methane oxidation (*pmoA*) and polyhydroxyalkanoate (PHA) biosynthesis (*phaABC*). Deciphering the genome will help research toward PHA production by MOB.

## ANNOUNCEMENT

**FIG 1:**
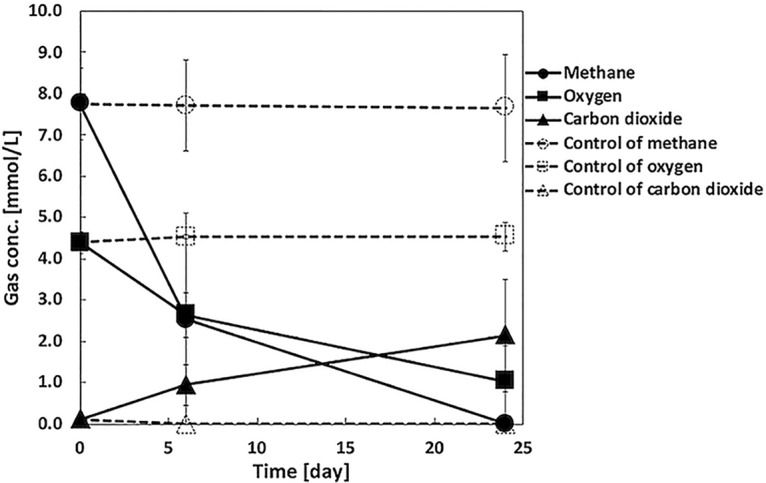
Methane oxidation activity of *Methylosinus* sp. strain C49 during batch culture. Solid circle, square, and triangle plots indicate methane, oxygen, and carbon dioxide concentrations, respectively, in the headspace. A control run without the cell suspension was operated in parallel to confirm the absence of unintentional methane consumption. Open circle, square, and triangle plots indicate methane, oxygen, and carbon dioxide concentrations, respectively, in the headspace. The experiment was conducted in triplicate.

*Methylosinus* sp. strain C49 was isolated from an enriched biomass with an inoculum from paddy field soil emitting highly concentrated methane from underground (1). The enriched biomass was subjected to dilution-extinction culturing with nitrate mineral salts medium (2). The grown cell suspension was diluted and spread onto gellan gum plates (3), followed by picking up of a grown colony for isolation. Subsequently, amplification of the 16S rRNA gene was performed to confirm the phylogeny as previously described (1). The isolate was identified as type II MOB genus *Methylosinus* using NCBI BLAST in blastn mode (4). The application of the cell suspension to the mixed gas (1:2 [vol/vol] ratio of methane and air) with the liquid medium (5) confirmed the methane oxidation activity by *Methylosinus* sp. strain C49 (Fig. 1).

The strain was incubated for 14 days at 30°C on a rotary shaker at 130 rpm with nitrogen mineral salts medium (2). The grown cells were harvested by centrifugation at 10,000 rpm for 5 min. The genomic DNA was extracted with a phenol-chloroform extraction technique (6), and cetyltrimethylammonium bromide was used for further purification. RNA as a contaminant in the genomic DNA was decomposed by RNaseA (TaKaRa Bio, Inc., Shiga, Japan). Regarding long-read sequencing, the library was prepared by using a one-dimensional (1D) ligation sequencing kit (SQK-LSK-109; Oxford Nanopore Technologies Ltd., Oxford, UK), without a fragmentation procedure, and sequenced on the MinION Mk1B instrument using an R.10 flow cell (FLO-MIN110; Oxford Nanopore Technologies Ltd.). The sequence data were base called using Guppy ver. 3.3.2 (7) with the high-accuracy mode. The attained sequence quality was confirmed using NanoPlot (ver. 1.20.0) (8), where the adaptor sequences, low-quality reads (Q < 7), header (75 bp), and short reads (<1,000 bp) were removed using Porechop ver. 0.2.4 (https://github.com/rrwick/Porechop) as a tool for trimming adapters. Regarding a short-read sequencing procedure, the MGIEasy universal DNA library prep set (MGI Tech, Shenzhen, China) was used for the library preparation according to the manufacturer’s protocol. Subsequently, 150-bp paired-end sequencing was performed with DNBSEQ-G400 (MGI Tech.) by a sequencing service (Bioengineering Lab. Co. Ltd., Kanagawa, Japan). The adapter sequences and low-quality reads (Q < 30) were removed using Trim Galore ver. 0.6.5 (http://www.bioinformatics.babraham.ac.uk/projects/trim_galore/). The consensus sequence was assembled using Unicycler ver. 0.4.7 (9) as a hybrid of the long and short reads. The genome completeness (97.5%) was assessed using BUSCO ver. 1 (10), and the missing marker gene (COG0088) was detected in the genome with BLASTn, ensuring the 100% completeness. The coding region of a gene was detected and annotated using DFAST ver. 1.1.5 (11) and KofamKOALA ver. 2020-01-06 (12), respectively. Default parameters were used for all software unless otherwise specified. The obtained genomic information is listed in Table 1.

**TABLE 1:** Genomic information of *Methylosinus* sp. strain C49

Type	Name	GenBank RefSeq accession no.	GenBank nucleotide accession no.	Size (Mb)	GC %	Contig length (bp)	Total avg depth (×)	No. of rRNAs	No. of tRNAs	No. of other RNAs	No. of genes	No. of pseudogenes
Chromosome		NZ_AP022332.1	AP022332.1	3.92	65.2	3,918,175	256	6	49	4	3,702	43
Plasmid	pMSC49a	NZ_AP022333.1	AP022333.1	0.32	64.6	324,889	161	3	3		205	6
Plasmid	pMSC49b	NZ_AP022334.1	AP022334.1	0.26	61.7	263,145	142				246	36
Plasmid	pMSC49c	NZ_AP022335.1	AP022335.1	0.18	62.8	178,244	99				163	13
Plasmid	pMSC49d	NZ_AP022336.1	AP022336.1	0.02	60.2	22,482	82		1		25	

Based on the gene-mapping to reference pathways of KEGG (release 89.1) (13), *Methylosinus* sp. strain C49 harbors the *pmoA* functional gene encoding methane monooxygenase capable of oxidizing methane to methanol. In addition, the *phaA*, *phaB*, and *phaC* genes encoding acetyl-coenzyme A (CoA) acetyltransferase, acetoacetyl-CoA reductase, and poly(3-hydroxyalkanoate) polymerase, respectively, essential for polyhydroxyalkanoate (PHA) biosynthesis, were found in the genome sequence.

### Data availability.

The complete genome sequence of *Methylosinus* sp. strain C49 has been deposited as five contigs in DDBJ/EMBL/GenBank under the accession numbers AP022332, AP022333, AP022334, AP022335, and AP022336. The BioSample accession number is SAMD00201571. The MinION read data were deposited in the DDBJ Sequence Read Archive (SRA) under BioProject accession number PRJDB9231 and SRA experiment accession number DRX196004 (run number DRR205605). DNBSEQ-G400 read data were deposited in the DDBJ Sequence Read Archive (SRA) under SRA experiment accession number DRX196005 (run number DRR205606).
